# Machine-intelligence for developing a potent signature to predict ovarian response to tailor assisted reproduction technology

**DOI:** 10.18632/aging.203032

**Published:** 2021-05-17

**Authors:** Sisi Yan, Wenyi Jin, Jinli Ding, Tailang Yin, Yi Zhang, Jing Yang

**Affiliations:** 1 Reproductive Medical Center, Renmin Hospital of Wuhan University and Hubei Clinic Research Center for Assisted Reproductive Technology and Embryonic Development, Wuhan 430060, China; 2 Department of Orthopedics, Renmin Hospital of Wuhan University, Wuhan 430060, China

**Keywords:** machine learning, prediction model, assisted reproduction technology, poor ovarian response

## Abstract

The prediction of poor ovarian response (POR) for stratified interference is a critical clinical issue that has received an increasing amount of recent concern. Anthropogenic diagnostic modes remain too simple for the handling of actual clinical complexity. Therefore, this study conducted extensive selection using models that were derived from a variety of machine learning algorithms, including random forest (RF), decision trees, eXtreme Gradient Boosting (XGBoost), support vector machine (SVM), and artificial neural networks (ANN) for the development of two models called the COS pre-launch model (CPLM) and the hCG pre-trigger model (HPTM) to assess POR based on different requirements. The results demonstrated that CPLM constructed using ANN achieved the highest AUC result of all the algorithms in COS pre-launch (AUC=0.859, C-index=0.87, good calibration), and HPTL constructed using random forest was found to be the most effective in hCG pre-trigger (AUC=0.903, C-index=0.90, good calibration). It is notable that CPLM and HPTM exhibited better performance than common clinical characteristics (0.895 [CPLM], and 0.903 [HPTM] in comparison to 0.824 [anti-Müllerian hormone (AMH)], and 0.799 [antral follicle count (AFC)]). Furthermore, variable importance figure elucidated the values of AMH, AFC, and E_2_ level and follicle number on hCG day, which provides important theoretical guidance and experimental data for further application. Generally, the CPLM and HPTM can offer effective POR prediction for patients who are receiving assisted reproduction technology (ART), and has great potential for guiding the clinical treatment of infertility.

## INTRODUCTION

As assisted reproduction technologies (ART) has advanced, the improvement of the clinical pregnancy rate has remained both a high priority and significant difficulty for fertility doctors [1]. Meanwhile, the response to controlled ovarian stimulation (COS) during ART is highly diverse and ovarian response plays crucial roles during this process [2]. In particular, poor ovarian response (POR), generally refers to a poor response to gonadotropin stimulation and is characterized by a low number of growing follicles which may result in poor oocyte retrieval, cycle cancellation, or even a failed reproductive outcome [3–5].

It is quite promising that researchers have discovered the advanced identification of poor responders to be of potential help in providing patients with more directed counseling which can lessen the disappointment of undesirable outcomes [6]. Generally, predicting POR before COS may be a contributor to formulating individualized programs [7], and prediction before hCG trigger day can facilitate the adjustment of trigger protocols (for example, when POR is predicted, GnRH-a + hCG double trigger [8, 9] can be used for the amelioration of IVF outcomes). These findings inspired us to predict POR based on clinical data in COS pre-launch and hCG pre-trigger in order to offer sufficient decision support.

Several clinically predictive indicators associated with POR have already been detected, such as age, basal follicle stimulating hormone (FSH), antral follicle count (AFC), and anti-Müllerian hormone (AMH) [10–13]. Significant attention has been paid to the comprehensive analysis of various indicators [14–16], but with current POR assessment approaches, traditional logistic regression is highly subjective and time-consuming [17], and is also unable to exploit interconnections between predictors and combinations of factors which may not be significant individually. Machine learning algorithms can be used for analyzing interactions between the exploratory variables of large data sets without knowledge of the form of the specific parameter function underlying the relationship [18]. Furthermore, many classical algorithms have been widely applied in ART, such as logistic regression (LR) [19] and machine learning, including decision tree [20], support vector machine (SVM) [21], and artificial neural network (ANN) [22, 23]. However, very few works have reported machine-learning models for the prediction of ovarian response, therefore, further exploration of the prediction potential of machine-learning algorithms in related fields was warranted.

In this study, the clinical data of patients undergoing IVF/ICSI was analyzed in order to establish optimum models for POR prediction (COS pre-launch model [CPLM] and hCG pre-trigger model [HPTM]) using different algorithms (typical statistical methods and machine learning models). By using these models, it was inferred that clinicians can apply appropriate therapeutic strategies mentioned above to infertile couples in order to increase the probability of favorable IVF outcomes.

## MATERIALS AND METHODS

### Data processing

The clinical data of 1,110 infertile women who had undergone IVF/ICSI treatment for the first time between July 2018 and May 2019 in Renmin Hospital of Wuhan University was retrospectively analyzed. Women with several different infertility factors were incorporated in order to establish a universal approach for POR prediction at our center.

### Patients’ characteristics and main outcomes

In the prospective cohort analysis, the main outcome measure was POR, which was defined as the retrieval of four or fewer oocytes or cycle cancellation [24]. Variables with a potential relationship to ovarian response were incorporated into our research, and models were constructed based on the various therapeutic stages of the treatment cycle:

(1) Variables of COS pre-launch model: age, BMI, infertility cause, infertility duration, infertility type, AMH, basic hormone levels (E_2_, FSH, and LH), AFC, pelvic surgery, and gravidity history.

(2) Variables of hCG pre-trigger model: all factors of the COS pre-launch model, plus therapeutic regimen, dosage of Gn (recombinant human follicle-stimulating hormone for injection, Gonal-f, German Merck Serono), days of Gn, E_2_ level on hCG day, and follicle number on hCG day (follicles with a diameter of ≥ 14 mm in bilateral ovaries).

### Feature selection

**Figure 1:**
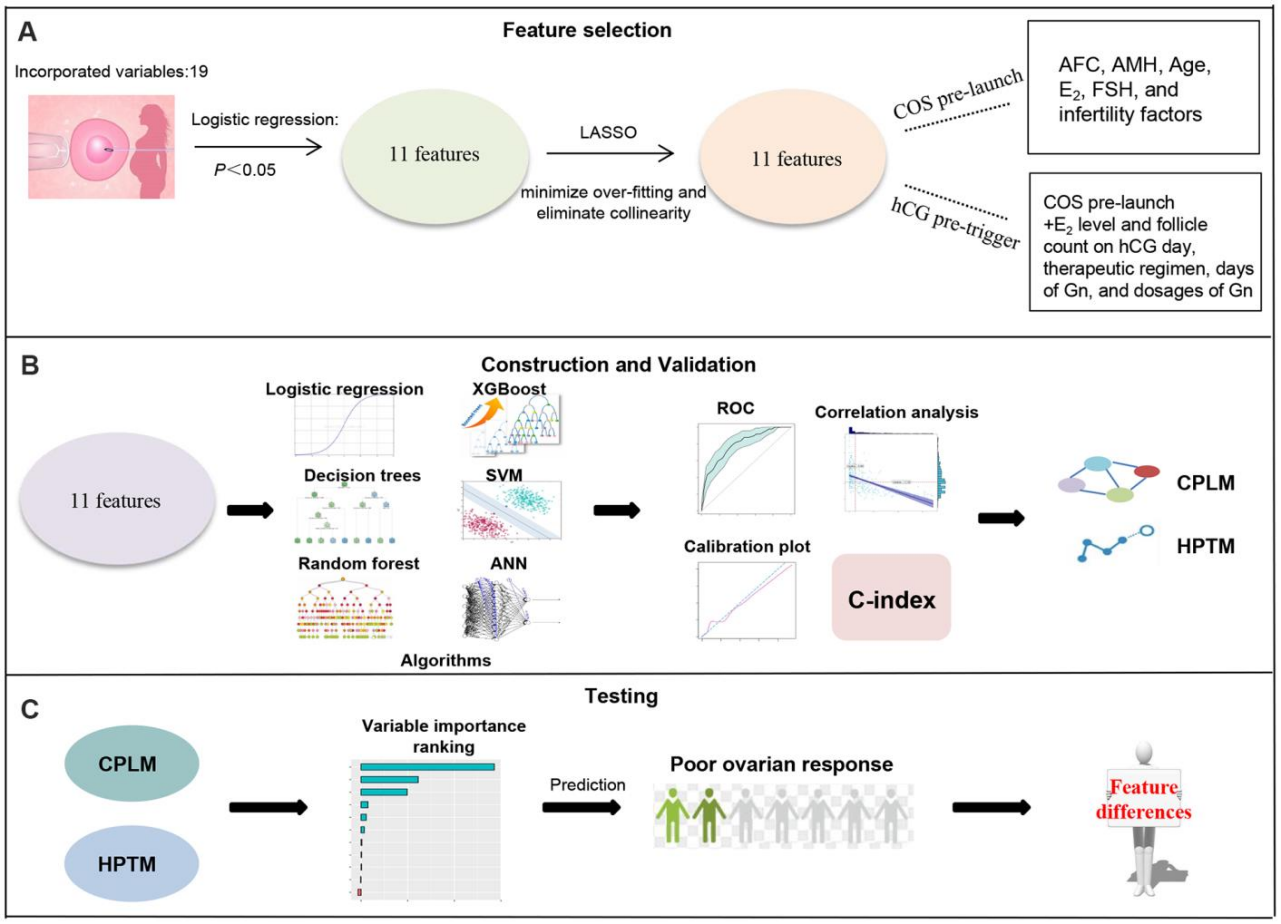
**Schematic workflow for poor ovarian response prediction.** (**A**) 11 features relating to ovarian response were obtained following logistic regression and LASSO. (**B**) These 11 candidate features were analyzed using multivariable logistic regression and machine learning, and then validated using ROC, calibration plot, C-index and correlation analysis to conduct CPLM and HPTM. (**C**) Variable importance of CPLM and HPTM were described to further understand and investigate of the models.

EpiData 3.1 software was used for establishing a database, and this was double-entered and validated by two qualified personnel. Once it was checked, the data was transferred to R software (version 3.6.4) and parameters proven to have a direct effect on ovarian response were screened using logistic regression; variables with a *P* < 0.05 were chosen for further analysis. After the effects of features on outcomes were fully assessed, least absolute shrinkage and selection operator (LASSO) regression was used for further minimization of the risk of over-fitting, and variables with high collinearity were eliminated. The LASSO regression was dependent on a cyclical coordinate descent algorithm and was conducted using a glmnet package in R software. The workflow of the study is presented in Figure 1.

### Construction of model

All data was randomly divided into a training dataset (70%) for feature selection and model training, and an independent validation dataset (30%) for repeated optimization and verification of the prediction model. And the models were set to use default parameters in R software.

### Multivariable logistic model

Normality was evaluated using a Kolmogorov-Smirnov test and Spearman’s Rho (nonparametric), or Pearson’s (parametric) bivariate correlation analysis was completed as deemed appropriate. For independent variables selected for the generalized multivariable logistic model, stepwise Akaike information criterion (AIC) was applied for eliminating multicollinearity and for selection of the model with the lowest AIC as the final model. A multivariate logistic model was also used to construct the ovarian response predictive model (ORPM). To facilitate this, the risk score was calculated using the following formula:

$$\text{Risk}\;\text{score}=\sum_{i=1}^{n}\beta_{i}\times E_{i}$$

where the risk score defined as ORPM-based risk signature was calculated by the ORPM - n represents the total number included in the ORPM, *β _i_* represents the regression coefficient of feature *i*, and *E _i_* refers to the coefficient of feature *i* in the constructed model.

### Machine learning

#### Decision tree

Decision tree algorithms use the Gini index to measure each decision point and create an optimal separation of the independent variables [25]. A dataset which minimizes the Gini index was selected after division as the optimal distribution in the subset of data. This splits the data which exhibited the best optimization criteria (subject to tree depth (11)) on our predictor.

#### Random forest (RF)

RF combines multiple decision trees and randomizes and summarizes the use of variables and data [26]. This study conducted RF containing 1,000 trees, where the maximum depth of each tree was determined based on the final numbers of the included features.

#### eXtreme gradient boosting (XGBoost)

XGBoost introduces the gradient descent algorithm and minimizes the loss when a new model is added, which helps it continuously learn a new function matching the residual of the previous prediction [27]. Similarly, XGBoost served as iterative model before reaching 1,000 cycles, and the maximum depth of each tree was determined based on the final numbers of the included features.

#### Support vector machines (SVM)

The aim of SVM is the establishment of a classification hyperplane that can correctly classify each sample and make the largest possible distance between the sample closest to the hyperplane for each sample type and the hyperplane [28].

#### Artificial neural network

ANN consists of an input layer, an output layer and one or more hidden layers between the input and the output layers. The most outstanding representative of the algorithm is resilient backpropagation learning [29]. In a typical process, hidden layers are determined to refer to the actual status, and the threshold is set as 0.005, the learning rate is set as 0.1, and parameter optimization is performed using rprop+ method.

### Validation of COS pre-launch model (CPLM) and hCG pre-trigger model (HPTM)

Several different approaches were utilized for the assessment of all models’ stratification abilities. Area under curve (AUC) was calculated from the receiver-operating characteristic (ROC) curve and was used to estimate the discrimination of each model. The accuracy of the derived models was evaluated by calibration plot, and models which shared a high goodness of fit with the dotted line were regarded as providing good calibration [30]. Notably, the net-classification index (NRI) was used to quantify the improvement of the predictive abilities of each model. The models with the highest ovarian response prediction accuracy in COS pre-launch and hCG pre-trigger models were defined as CPLM and HPTM. The contribution and importance of each CPLM/HPTM-based signature were quantified using mean concordance-index (C-index). Spearman correlation analysis was then performed to accurately determine the correlation between the CPLM and HPTM scores of each patient and the corresponding retrieved oocytes.

### Grouped analysis for potential difference of clinical features

Statistical comparisons of patients’ clinical characters were performed using Wilcoxon’s test, and *P*-value adjustment using the Benjamini-Hochberg procedure.

### Statistics

R software (version 3.6.4) was used for data processing and analysis.

### Ethics approval and consent to participate

Written informed consent was obtained from each participant and the study was approved by the ethical committee of the Renmin Hospital of Wuhan University.

## RESULTS

### Demographic and clinical characteristics of participants

Based on the number of oocytes retrieved, the prevalence of POR was 14.59% in the present cohort. The demographic parameters of participants are displayed in Table 1. Poor ovarian responders were older than the normal to high responders, and exhibited significantly higher E_2_, FSH, days of Gn, dosages of Gn, E_2_ level and follicle number on hCG day. Significantly differences were also presented regarding infertility cause and therapeutic regimen.

**Table 1:** Baseline participant characteristics.

**Parameters**		**Normal to high responders**	**Poor responders**	**P**	**Effect size**
E_2_ level on hCG day		3090.00(2295.00-4539.00)	1422.20(799.80-2267.80)	<0.001	*r* = 0.396
follicle number on hCG day		12.00(8.00-16.25)	5.00(3.00-8.00)	<0.001	*r* = 0.402
AFC		16.00(11.00-21.00)	9.00(5.00-13.00)	<0.001	*r* = 0.365
AMH		3.09(1.99-5.05)	1.03(0.56-1.75)	<0.001	*r* = 0.420
infertility duration		3.00(2.00-6.00)	4.00(2.00-6.00)	0.297	*r* = -0.031
infertility cause	pelvic and fallopian tube factors	347(36.60)	51(31.48)	<0.001	*V* = 0.211
	ovulatory obstacle	74(7.81)	1(0.62)		
	endometriosis and uterine factors	42(4.43)	19(11.73)		
	decreased ovarian reserve	147(15.50)	32(19.75)		
	unexplained infertility	64(6.75)	22(19.75)		
	male factor	175(18.46)	20(12.35)		
	multiple confounding factors	99(10.44)	17(10.49)		
therapeutic regimen	long protocol	430(45.36)	51(31.48)	<0.001	*V* = 0.268
	super-long protocol	263(27.74)	21(12.96)		
	antagonist regimen	162(17.09)	34(20.99)		
	PPOS	87(9.18)	54(33.33)		
	others	6(0.63)	2(1.23)		
age		30.0(28.0-33.0)	32.00(29.00-36.00)	<0.001	*r* = -0.146
E_2_		35.60(31.45-41.99)	49.31(37.03-55.77)	<0.001	*r* = -0.303
FSH		6.870(5.707-8.210)	8.315(6.798-10.515)	<0.001	*r* = -0.217
LH		3.645(2.730-4.832)	3.515(2.525-4.562)	0.125	*r* = 0.046
days of Gn		10.00(9.00-11.00)	10.000(8.000-11.000)	<0.001	*r* = 0.116
dosages of Gn		2250(1725-2900)	2700(2025-3200)	<0.001	*r* = -0.117
pelvic surgery	No	718(75.74)	120(74.07)	0.649	*r* = 0
	Yes	230(24.26)	42(25.93)		
gravidity history	0	484(51.05)	77(47.53)	0.522	*r* = 0
	1	236(24.89)	41(25.31)		
	2	116(12.24)	21(12.96)		
	≥3	112(11.82)	23(14.19)		
height		160.0(158.0-163.0)	160.0(158.0-163.0)	0.904	*r* = 0.004
weight		56.00(51.00-62.00)	56.00(51.00-64.00)	0.525	*r* = -0.019
BMI		21.64(19.98-23.93)	21.95(20.20-24.23)	0.357	*r* = -0.028
infertility type	primary infertility	452(47.68)	72(44.44)	0.446	*r* = 0
	secondary infertility	496(52.32)	90(55.56)		

Normally distributed data, skewed distribution data and nominal data are described by mean ± SD, median ± interquartile range and frequency (relative frequency) respectively. Wilxon signed-rank test and chi-square test were applied in skew distribution data and nominal data respectively, and properly used *r* and *V* as the effect size to quantify the significance.

### Feature engineering

In order to prevent the risk of over-fitting and to screen the important features which impact outcomes for the optimization of the constructed models, feature engineering was conducted. LASSO regression combined with univariable logistic regression was performed to narrow the candidate features, the results of which were displayed in Table 2 and Supplementary Figure 1A, 1B. A total of 11 features remained of the original 19 features, and those selected were confirmed to be important regarding outcome. The significant variables identified following the selection procedure were recorded as follows: AFC, AMH, Age, E_2_, FSH, and infertility factors were incorporated in the COS pre-launch model. Variables in the hCG pre-trigger model included all factors from the COS pre-launch model, in addition to E_2_ level and follicle number on hCG day, therapeutic regimen, days of Gn, and dosages of Gn.

**Table 2:** Odds ratio and *p*-values calculated from the univariable logistic regression for quantifying the impacts of parameters included in the present study on ovarian response.

**Parameters**	**Odds ratio (95% CI)**	***p***
E_2_ level on the hCG day	1.00 (1.00-1.00)	5.97E-27**
follicle number on hCG day	0.75 (0.71-0.79)	1.34E-29**
AFC	0.81 (0.78-0.84)	4.91E-27**
AMH	0.37 (0.30-0.44)	1.92E-25**
infertility years	1.03 (0.98-1.08)	0.274
infertility cause	1.09 (1.01-1.17)	0.025*
therapeutic regimen	1.71 (1.47-1.98)	1.44E-12**
age	1.10 (1.06-1.14)	4.79E-07**
E_2_	1.07 (1.06-1.09)	3.82E-22**
FSH	1.25 (1.18-1.33)	5.43E-13**
LH	0.94 (0.85-1.03)	0.222
days of Gn	0.88 (0.82-0.95)	0.001026*
dosages of Gn	1.00 (1.00-1.00)	7.20E-05**
pelvic surgery	1.09 (0.74-1.59)	0.649
gravidity history	1.01 (0.89-1.14)	0.815
height	0.99 (0.96-1.03)	0.684
weight	1.00 (0.99-1.02)	0.614
BMI	1.02 (0.97-1.07)	0.440
infertility type	1.14 (0.82-1.60)	0.446

*, *P*<0.05; **, *P*<0.01.

### Construction and comparison of method performance

After the process of feature selection completed, statistic models and machine-learning models were trained and validated according to the aforementioned methods. For COS pre-launch models, parameters of logistic model and decision tree were represented in Table 3 and Supplementary Figure 3, respectively; and framework of other machine-learning models including RF, XGBoost, SVM, and ANN please visit our data online at https://data.mendeley.com/datasets/tpj39wptts/1. For hCG pre-trigger models, components of logistic model and decision tree were exhibited in Table 4 and Supplementary Figure 4, respectively; and framework of machine-learning models please visit our data online at https://data.mendeley.com/datasets/tpj39wptts/1.

**Table 3:** Coefficients of trained logistic regression for COS pre-launch models.

**Parameters**	**coefficients**	**Odds ratio (95% CI)**	***p***
AFC	-0.115	0.891(0.843-0.94)	<0.001**
AMH	-0.729	0.482(0.368-0.616)	<0.001**

*, *P*<0.05; **, *P*<0.01.

**Table 4:** Coefficients of trained logistic regression for hCG pre-trigger models.

**Parameters**	**coefficients**	**Odds ratio (95%CI)**	***p***
E_2_ level on the hCG day	-0.001	1.000(0.9999-1.000)	0.004*
follicle number on hCG day	-0.071	0.932(0.850-1.014)	0.115
AFC	-0.063	0.939(0.880-1.000)	0.054
AMH	-0.618	0.539(0.405-0.697)	< 0.001**
FSH	-0.077	0.926(0.848-1.009)	0.081
dosages of Gn	-0.001	1.000(1.000-1.000)	0.082

*, *P*<0.05; **, *P*<0.01.

**Figure 2:**
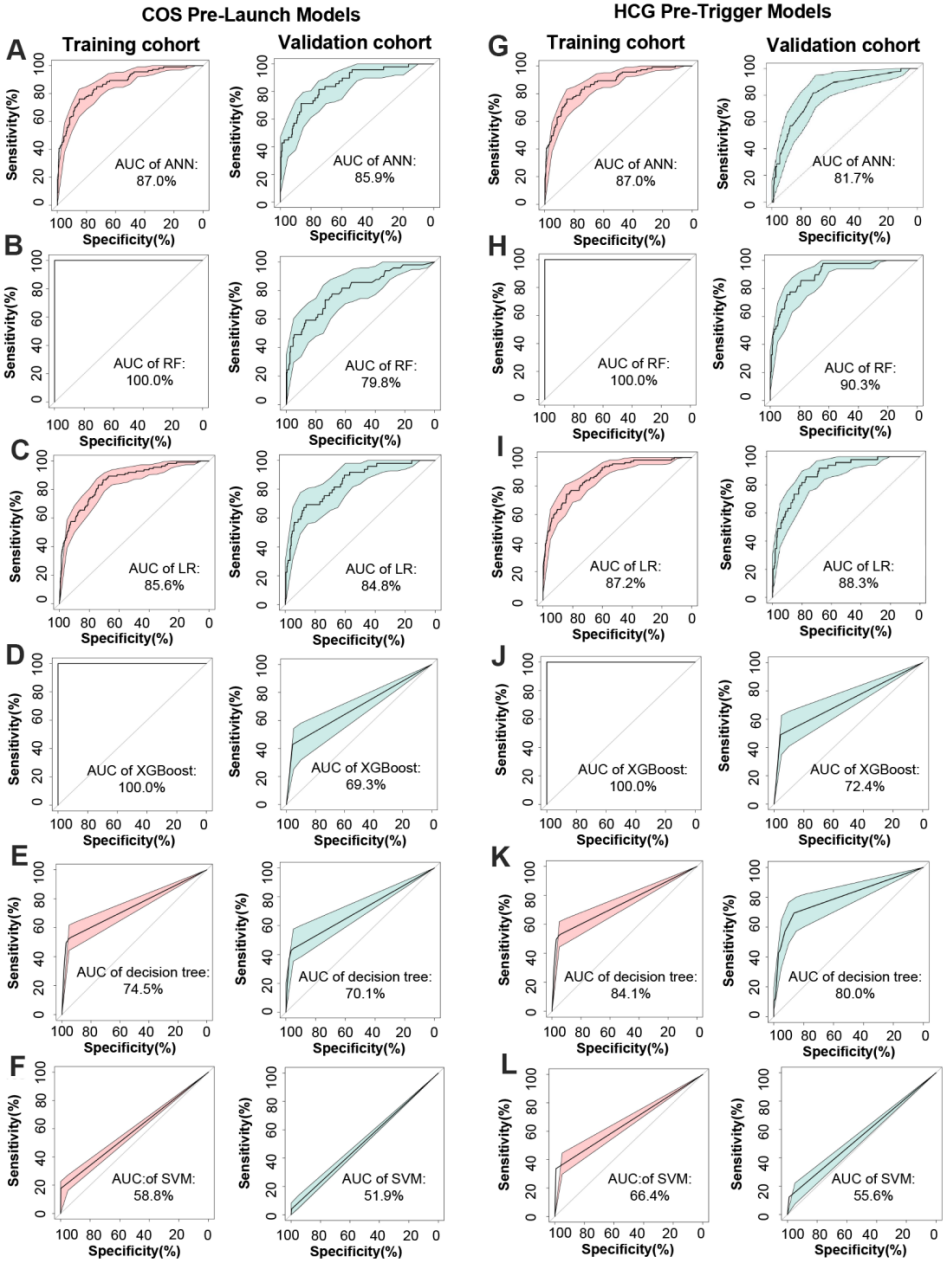
**Construction and comparison of method performance.** (**A**–**F**) ROC curve of ANN, RF, LR, XGBoost, decision tree, and SVM for target cohort in COS pre-launch models, respectively. (**G**–**L**) ROC curve of ANN, RF, LR, XGBoost, decision tree, and SVM for target cohort in hCG pre-trigger models.

It has been demonstrated that the area under the ROC curve (AUC) is a puissant indicator for the prediction of dichotomous outcomes, and then the AUC was examined to assess the accuracy of the constructed models. As can be seen in Figure 2A–2L, ANN yielded optimum predictive ability and accuracy in all algorithms with an AUC of 0.859 in COS pre-launch models, and the RF had the highest AUC (0.903) in hCG pre-trigger models. The predictive ability and accuracy of logistic regression (AUC = 0.848 and 0.883 corresponded to COS pre-launch and hCG pre-trigger models) and decision tree (AUC= 0.701 and 0.800) were slightly worse in comparison to ANN or RF. XGBoost produced relatively poor results with AUC of 0.724 and 0.693, and SVM exhibited the minimum prediction efficiency, with AUC of 0.556 and 0.519. Similar trends were also observed in the training cohort.

### Validation of CPLM and HPTM

As they have been proven to be the best models for the estimation of ovarian response, derived ANN and RF models were considered as CPLM and HPTM and further investigations were conducted. C-index was determined for reaffirming CPLM and HPTM prediction accuracy. After 1,000 estimations were made using the bootstrap method, the mean C-index of the validation cohort’s CPLM and HPTM were 0.87 and 0.90, respectively. This demonstrated that the predicted results for CPLM and HPTM were highly consistent with the actual value, and represented high accuracy among the constructed models [31]. The training cohort also demonstrated similar results regarding C-index.

**Figure 3:**
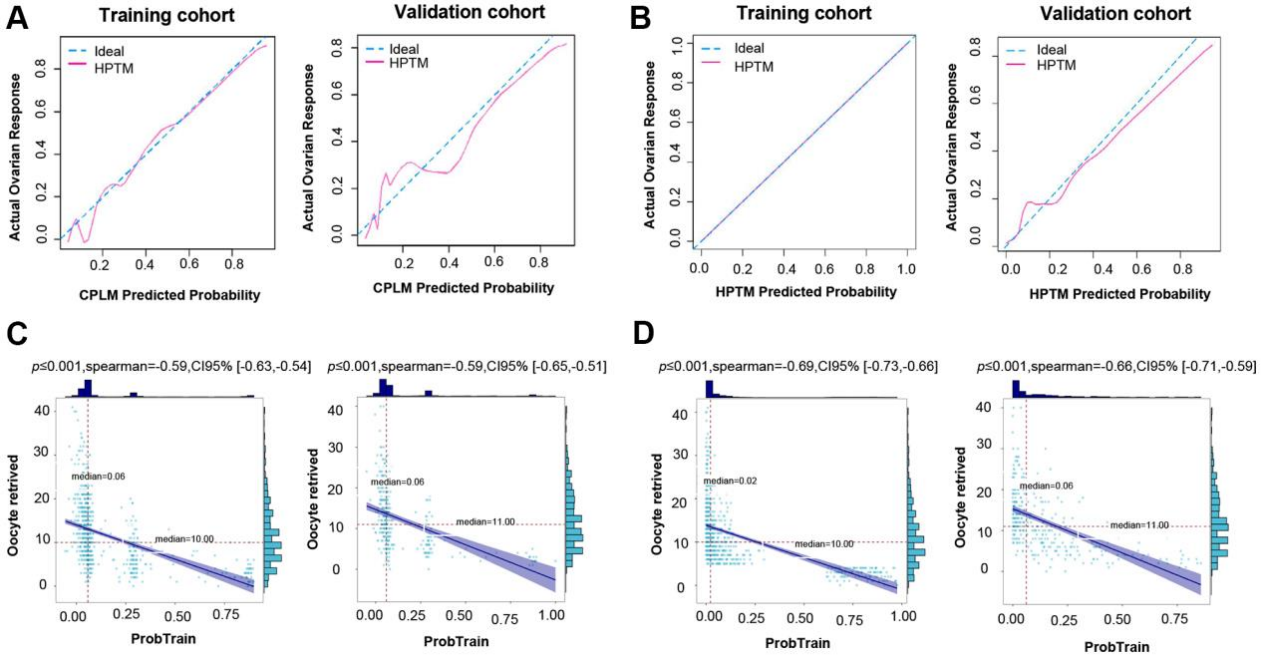
**Validation of CPLM and HPTM.** (**A**) The calibration plot for training and validation data was used to analyze the consistency of poor ovarian response between the predicted value and the observed proportion in CPLM. (**B**) The calibration plot for training and validation data was used to analyze the consistency of poor ovarian response between the predicted value and the observed proportion in HPTM. (**C**) Correlation analysis of the CPLM score and relevant retrieved oocytes in the cohort. (**D**) Correlation analysis of the HPTM score and relevant retrieved oocytes in the cohort.

In addition, a calibration plot measuring calibration ability also showed that the predicted value of the CPLM and HPTM-based signature was in accordance with the observed proportion (Figure 3A, 3B).

For further evaluation of the model’s credibility, correlation analysis between the CPLM and HPTM scores and the corresponding number of retrieved oocytes for each patient were determined. The analysis results demonstrated that each patient’s CPLM and HPTM scores were correlated negatively with retrieved oocytes, thereby suggesting that the retrieved oocytes gradually decreased as the score increased (Figure 3C, 3D), and the relevant correlation coefficient was 0.59 and 0.69 in CPLM and HPTM, respectively.

All aforementioned evidence was presented following a series of investigations, which strongly indicated that the constructed models reached an optimum contribution and employed a small enough number of clinical characters without losing their predictive value.

### Comparison between CPLM/HPTM and common clinical characteristics

**Figure 4:**
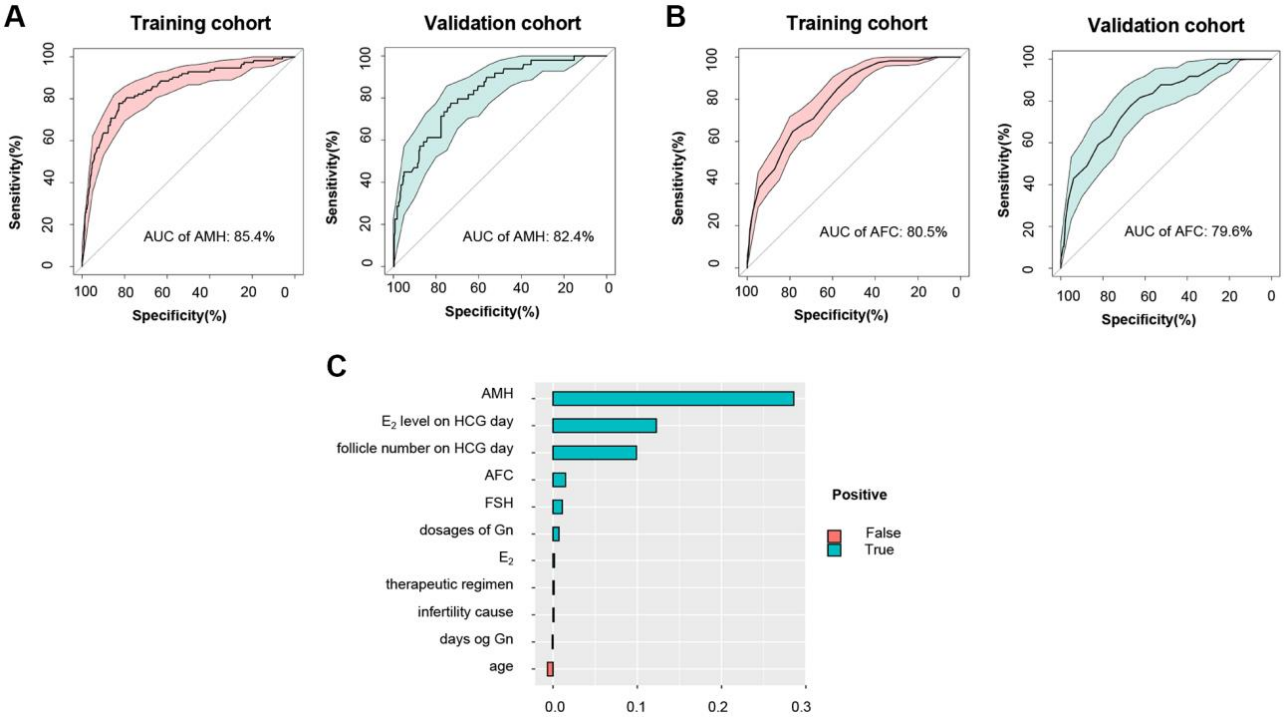
**Comparison between HPTM and common clinical characteristics.** (**A**) ROC curve and the corresponding AUC of AMH for training and validation cohort. (**B**) ROC curve and the corresponding AUC of AFC for training and validation cohort. (**C**) Variable importance ranking in HPTM.

Numerous studies have proven AMH and AFC to be the most effective parameters for the prediction of poor ovarian response in ART [4, 32]. An evaluation of the effectiveness of obtained CPLM and HPTM was performed through a comparison of the above characteristics to establish both their superiority and applicability in clinical practice. The results were encouraging and revealed that the AUC of CPLM and HPTM (0.903 and 0.859) (Figure 2A, 2H) were superior to those of the most common clinical characteristics - AMH and AFC (0.824 and 0.796) (Figure 4A, 4B), indicating that the constructed models had more valuable prediction signatures than common clinical characteristics.

NRI is a method for measuring a model’s accuracy based on changes made to the number of correct classifications. Results showed that CPLM had better accuracy compared to AMH and AFC (NRI =13.4% and 18.8%, respectively). In addition, HPTM’s accuracy was considerably higher than that of AMH and AFC (compared to AMH, NRI = 74.7%; compared to AFC, NRI = 82.6%), and CPLM and HPTM’s prediction efficiency was preferable. Similar trends were observed in the training cohort (Table 5).

**Table 5:** NRI results of CPLM and HPTM when compared to AMH and AFC.

**Models**	**Cohort**	**AMH**	**AFC**
		**(95% CI)**	**(95% CI)**
CPLM vs.	Train	13.4%**	18.8%**
		(9.3%-14.2%)	(5.5%-33.1%)
	Validation	18.4%**	21.1%**
		(5.3%-19.1%)	(29.3%-49.6%)
HPLM vs.	Train	74.7%**	82.6%**
		(66.4%-83.0%)	(75.4%-89.8%)
	Validation	27.50%	37.7%**
		(11.4%-43.6%)	(21.8%-53.7%)

*, *P*<0.05; **, *P*<0.01.

### Variable importance ranking in CPLM and HPTM

For facilitation of the clinical decision process, variable importance figures of CPLM and HPTM were used to investigate the models. As can be seen in Supplementary Figure 2 and Figure 4C, AMH was the most important predictor for POR, conforming to findings of the latest study which emphasized the significance of AMH [33]. Indictors including AFC and FSH that are commonly used for the assessment of ovarian response also displayed significant contribution to the objective function. In addition, HPTM highlighted the illustrious positions of E_2_ level and follicle number on hCG day in the prediction of hCG pre-trigger model. However, age, dosages of Gn, E_2_, therapeutic regimen, and days of Gn were proven to be slightly less significant in the models.

### Potential differences between high- and low-risk group identified by CPLM or HPTM

**Figure 5:**
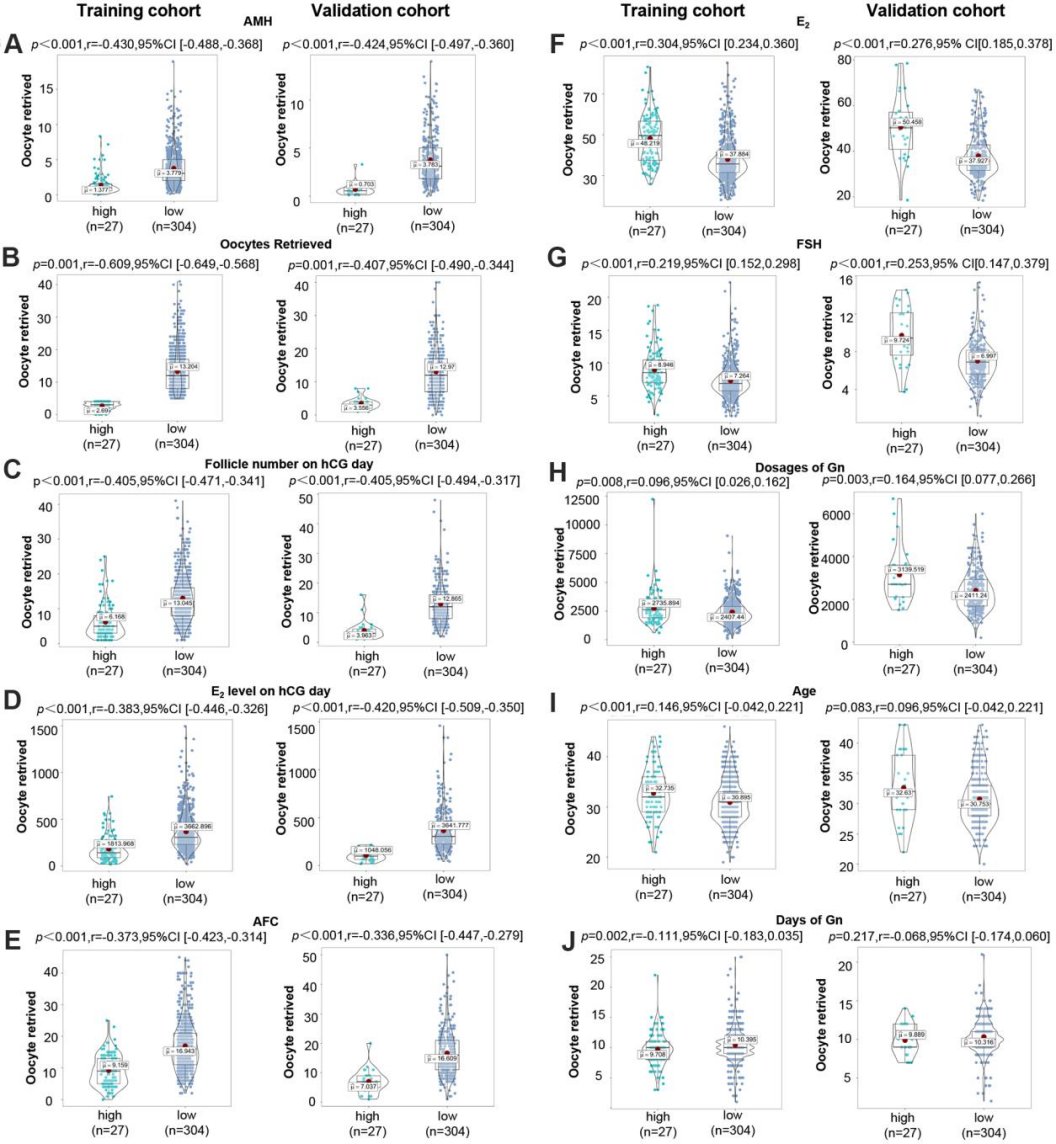
**Potential differences between high- and low-risk groups identified by CPLM or HPTM.** (**A**–**J**) Distribution of AMH, oocytes retrieved, follicle number on hCG day, E_2_ level on hCG day, AFC, E_2_, FSH, dosages of Gn, age, and days of Gn in the high-risk group and poor ovarian response in the high-risk group.

In order to detect potential differences in clinical characteristics between the high-risk group (with a higher risk of predicting to be POR) and the low-risk group defined by CPLM and HPTM, grouped analyses were performed. As is shown in Figure 5A–5J, significant differences were discovered between both groups, with the exception of age and days of Gn. The validation results were as follows: AMH (r=-0.424, *P*<0.001), oocytes retrieved (r=-0.407, *P*=0.001), E_2_ level on hCG day (r=-0.420, *P*<0.001), follicle number on hCG day (r=-0.405, *P*<0.001), AFC (r=-0.366, *P*<0.001), E_2_ (r=0.276, *P*<0.001), FSH (r=0.253, *P*<0.001), dosages of Gn (r=0.164, *P*=0.003). However, training results indicated that AMH (r=-0.430, *P*<0.001), oocytes retrieved (r=-0.609, *P*=0.001), E_2_ level on hCG day (r=-0.383, *P*<0.001), follicle number on hCG day (r=-0.405, *P*<0.001), AFC (r=-0.373, *P*<0.001), E_2_ (r=0.304, *P*<0.001), FSH (r=0.219, *P*<0.001), age (r=0.146, *P*<0.001), dosages of Gn (r=-0.096, *P*=0.008), and days of Gn (r=-0.111, *P*=0.002), all exhibited significant differences between both groups. The results presented above further prove the efficacy of CPLM and HPTM, and emphasize the value of AMH, E_2_ level on hCG day, follicle number on hCG day, AFC, E_2_, and FSH.

## DISCUSSION

This study has provided the first report for establishing CPLM and HPTM in the prediction of ovarian response at various therapeutic stages of IVF cycles using multiple machine learning algorithms, when individualized interference is available to sterile couples. This study was also the first attempt where machine learning was applied to routine medical practice to facilitate the improvement of clinical management and provide successful outcomes for infertile couples in ART.

One significant advantage of this study is the machine learning-based CPLM and HPTM, which can be implemented in related clinical processes for predicting ovarian response in sterile women, which will also allow the application of individualized stratified interference. Machine learning is based on non-linear parallel processing and has identified a new direction in the field of IVF, improving reason and self-organization, as it continues to learn [34, 35]. Several machine learning algorithms, including RF, decision tree, XGBoost, SVM, and ANN, were used in this research for the selection of two models in COS pre-launch and hCG pre-trigger, which were considered to be CPLM and HPTM. During this competition, where AUC was used as an evaluation indicator, an RF model as a CPLM, and an ANN model as an HPTM were excelled. More specifically, for CPLM selection, the ANN model demonstrated better prediction performance with an AUC value of 0.859, followed by LR, RF, decision tree, XGBoost, and SVM model (0.848, 0.798, 0.700, 0.693, and 0.519, respectively). Regarding HPTM selection, the RF model demonstrated excellent success with an AUC value of 0.903, followed closely by LR, decision tree, ANN, XGBoost, and SVM model (AUC=0.883, 0.841, 0.817, 0.724, and 0.556, respectively).

After screening of CPLM and HPTM, both models were characterized. For the assessment of prediction accuracy, the mean C-index of CPLM and HPTM were 0.87 and 0.90; both exhibiting excellent calibration properties. These findings indicated that the predicted results for CPLM and HPTM were highly consistent with the actual value, thereby representing a high level of accuracy in the constructed models. It is notable that machine learning-based CPLM and HPTM triumphed over the traditional statistical model (AUC 0.859 vs 0.848, for CPLM; 0.903 vs 0.883, for HPTM). Meanwhile, an independent validation dataset used in our research also verified the superiority of the constructed models. This evidence adequately demonstrates the advantages of employed machine learning algorithms, proving that they are highly effective models for predicting outcomes. To gain further clarification regarding the clinical applicability of CPLM and HPTM, both models were compared with AMH and AFC, which are the most commonly used clinical characteristics for ovarian response prediction. As anticipated, CPLM proved to be more effective for outcome prediction than AMH and AFC (0.868 vs 0.824 [AMH], and 0.796 [AFC]), as did HPTM (0.903 vs 0.824 [AMH], and 0.796 [AFC]). A previous meta-analysis using random intercept logistic regression demonstrated that AMH and AFC are both accurate ovarian response predictors. In this study, CPLM and HPTM proved to be more accurate than AMH and AFC, and other reported prediction models where AUC varied between 0.39 and 0.88 [14, 36–39]. These findings strongly demonstrate that there is great clinical application potential for this study’s constructed CPLM and HPTM due to the high accuracy they have for POR prediction.

To further evaluate the importance of the features incorporated in the chosen CPLM and HPTM, variable importance rankings were established. It is notable that both models displayed robust significance in AMH, AFC, and FSH, irrespective of different time periods, thereby indicating the important value of these traits during IVF concluded in previous researches [40–42]. It is of great significance that this study’s results were similar to those obtained through previous studies, which indicates that AMH with the highest variable importance value in CPLM and HPTM is the most important variable for POR prediction [43, 44]. Although age had previously been considered to be of great value for ovarian response prediction [45], several studies have placed more focus on “ovarian age”, and this study was consistent with them in demonstrating that age should not be regarded as a stable characteristic for POR prediction [14, 46, 47]. In addition, variable importance results in HPTM proved that both E_2_ levels and follicle number on hCG day play important roles, as E_2_ levels on hCG day can reflect the secretory function of follicles and they are related to the number and size of follicles in both ovaries during COS, which is considered to be a marker of ovarian reactivity [48]. Previous research has also demonstrated that E_2_ level on hCG day is an independent POR marker, which further highlights the importance of the indicator [49, 50]. It is of interest that days of Gn are associated with follicular maturation and appropriate extension of days of Gn can improve follicular maturation and retrieved oocytes [51]. Similarly, the models used in this study also attached significant importance to days of Gn, proving that clinicians should have greater focus on the individualized use of ovulatory drugs.

In this study, the prediction efficiency of HPTM was proven to be greater than that of CPLM. The main reason for this could be that HPTM incorporates additional important characteristics (E_2_ lever and follicle number on hCG day), which are particularly significant in ovarian response prediction [52, 53]. However, it is notable that HPTM is better suited to hCG pre-trigger in terms of delayed information. Accordingly, clinicians can access ovarian response based on the CPLM before treatment cycles for the formulation of individualized regimens, whereas HPTM can be used for guidance on hCG administration day.

This study was limited due to being retrospective regarding design and the data was obtained from only one fertility center. In addition, the models failed in the prediction of retrieved oocytes, embryo quality, or IVF outcomes. Therefore, long-term research with a greater, multicenter sample and a more in-depth exploration of IVF outcomes is required in order to provide confirmation of the efficacy of our findings.

## CONCLUSIONS

To summarize, the current study’s CPLM and HPTM exhibited higher accuracy for poor ovarian response prediction in sterile women than the reported models of AMH and AFC as clinical indicators. The constructed models used in this study can access more precise individualized interference for the implementation of related clinical processes which will help achieve better pregnancy outcomes.

### Data availability statement

All generated data was included in the present study.

## Supplementary Material

Supplementary Figures
