# Supplementary material for: Machine-intelligence for developing a potent signature to predict ovarian response to tailor assisted reproduction technology
Source: Aging (Albany NY). 2021 May 17;13(13):17137–54. doi: 10.18632/aging.203032 (PMC8312467; doi:10.18632/aging.203032)
Supplement: Supplementary Figures [file aging-13-203032-s001.pdf]

# SUPPLEMENTARY FIGURES

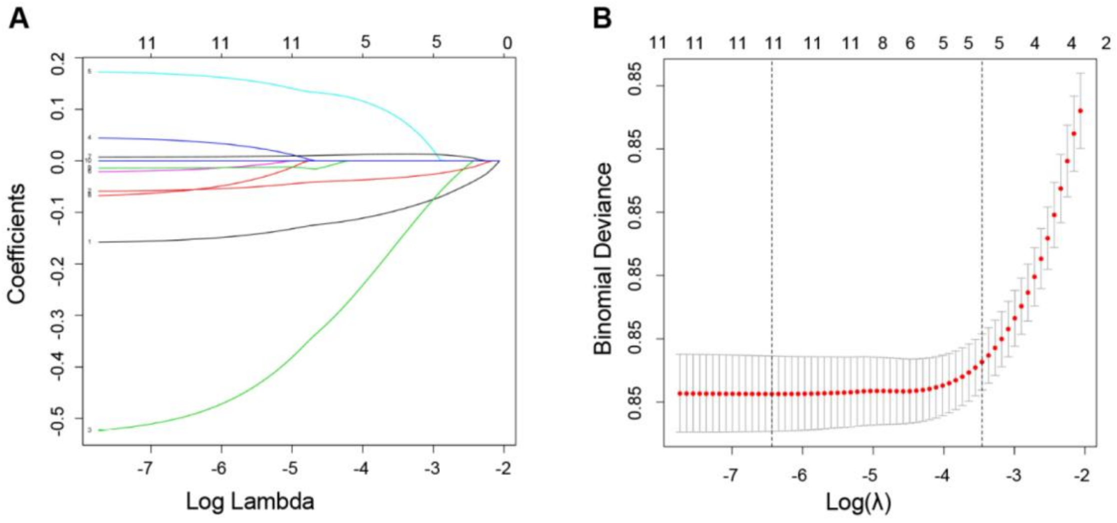


**Supplementary Figure 1. LASSO Cox regression model.** (**A**) Plot of LASSO coefficient profiles. (**B**) Plot of partial likelihood deviance for the 11 features in the study cohort.

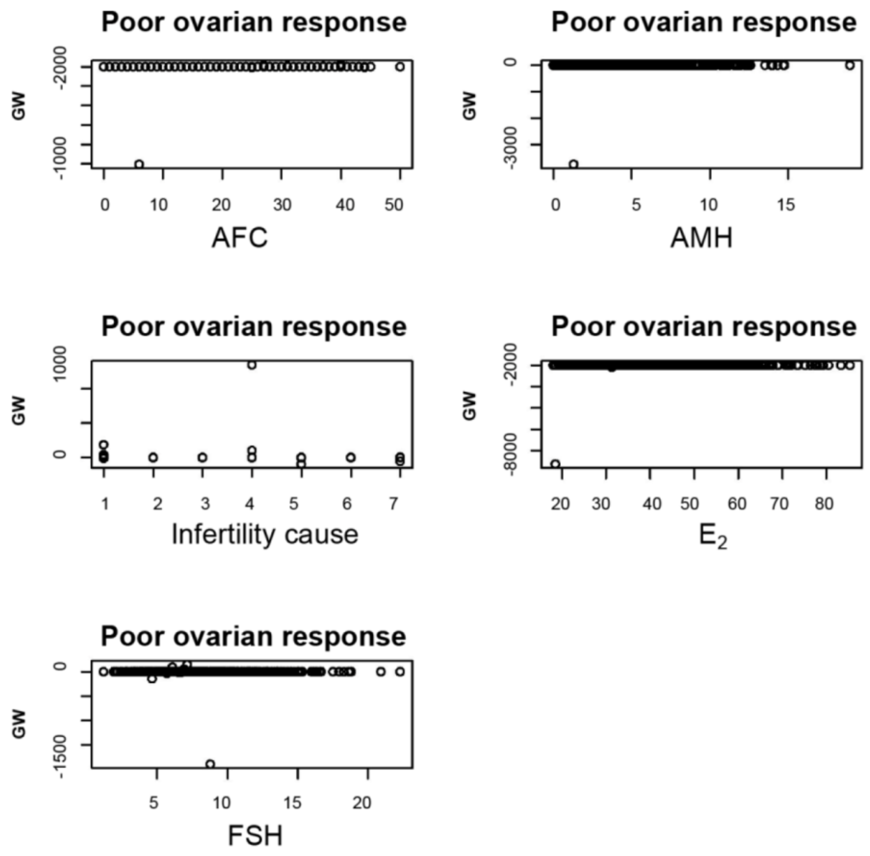


**Supplementary Figure 2. Variable importance ranking in CPLM.**

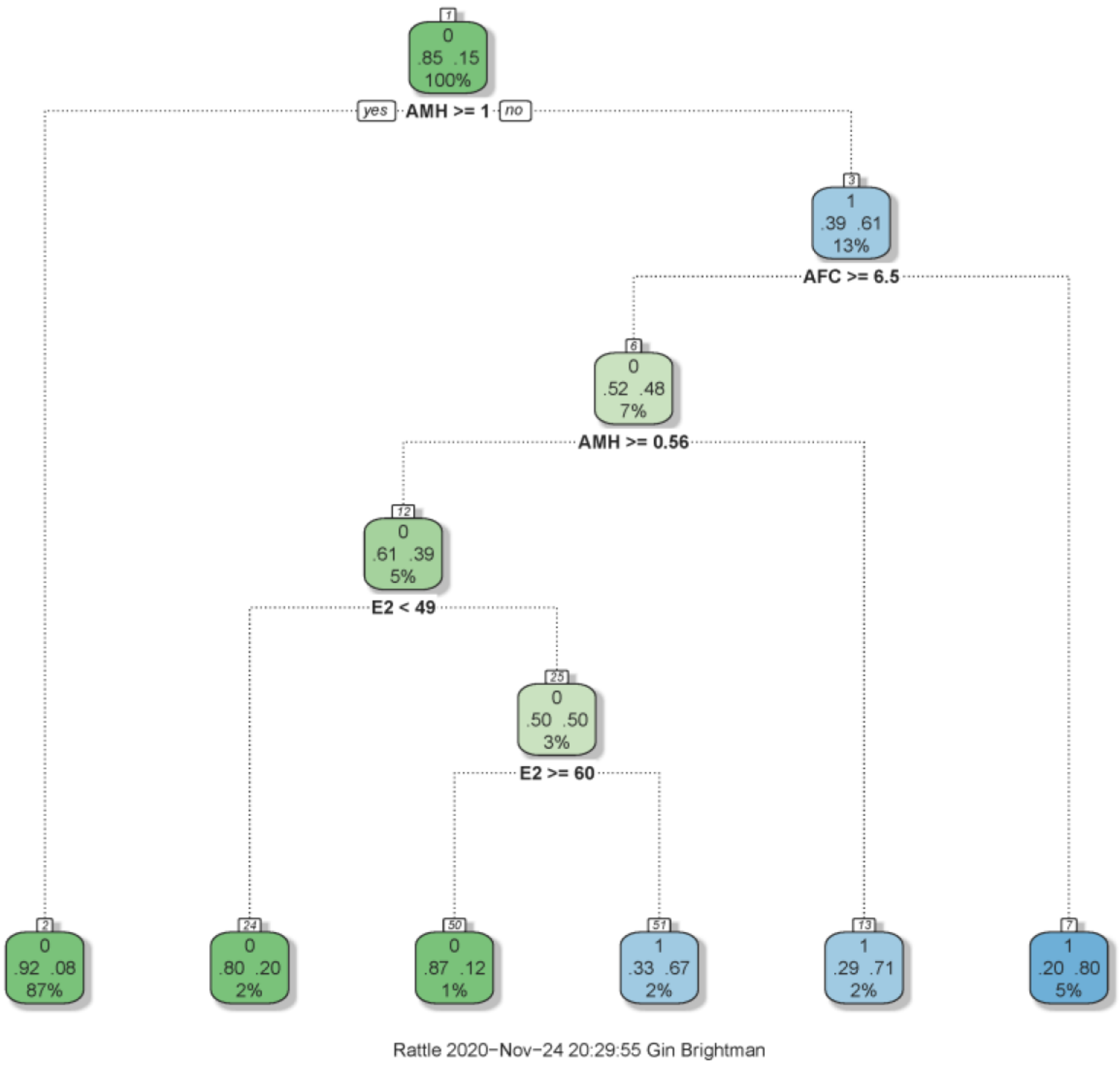


**Supplementary Figure 3. Parameters of decision tree in COS pre-launch models.**

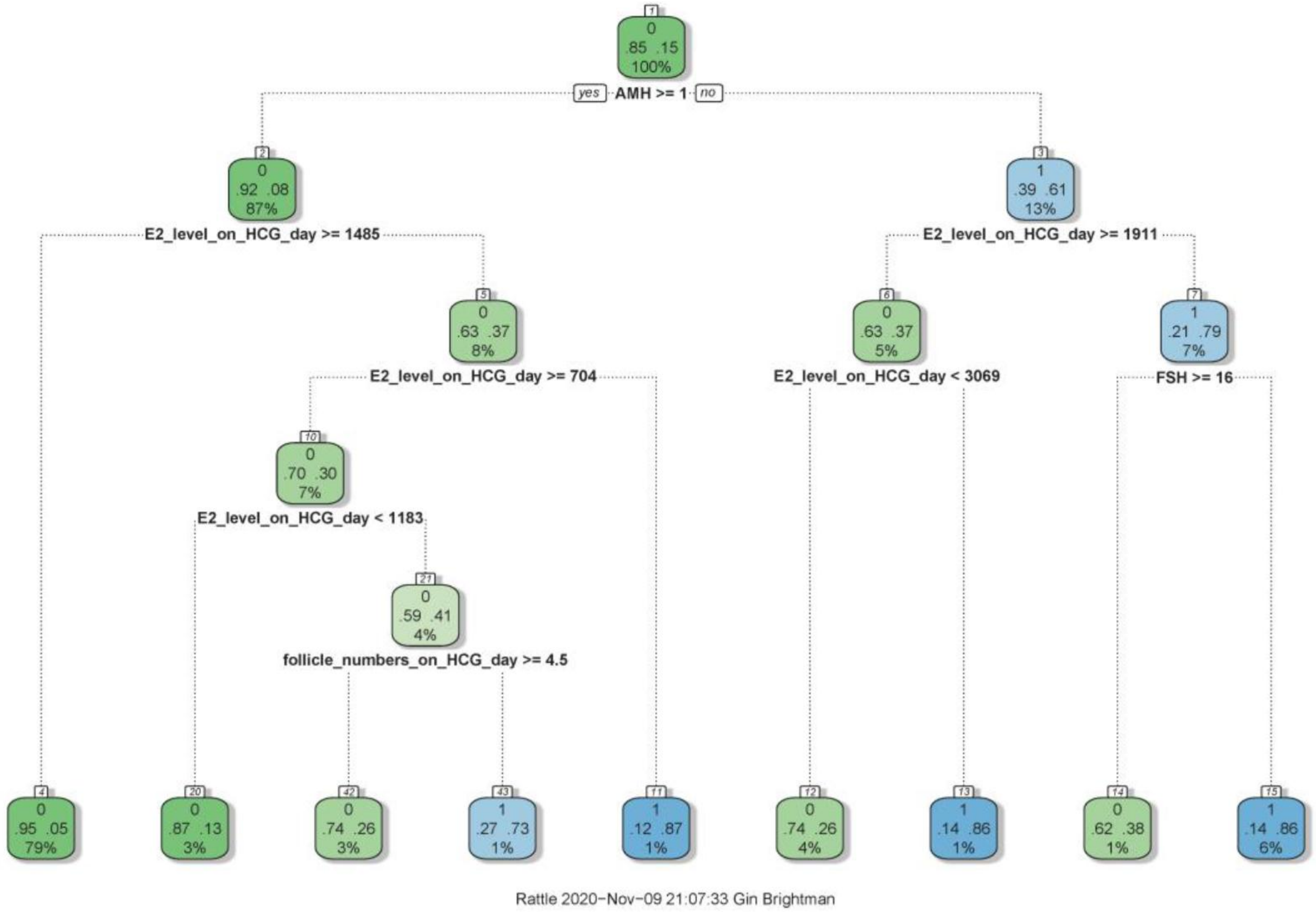


**Supplementary Figure 4. Parameters of decision tree in hCG pre-trigger models.**
